# Population Ecology and Harvesting of Rooibos ( *Aspalathus linearis* ) and Its Ecotypes in the Wild, South Africa

**DOI:** 10.1002/pei3.70079

**Published:** 2025-08-05

**Authors:** Tineke Kraaij, Gerhard C. P. Pretorius

**Affiliations:** ^1^ School of Natural Resource Science and Management, Faculty of Science, Nelson Mandela University George South Africa; ^2^ NaturaLibra Environmental Services CC Malmesbury South Africa

**Keywords:** biotrade species, fire frequency, plant allometry, population demographics, population health, resource status assessment, resource utilization, wild harvesting

## Abstract

*Aspalathus linearis* (‘rooibos’) is a polymorphic perennial shrub native to the drier, northwestern part of the Fynbos Biome in the Cape Floristic Region. It is cultivated on a large scale and wild‐harvested on a small scale to produce rooibos tea, a traditional herbal drink. Rooibos is a post‐fire pioneer germinating from fire‐stimulated soil‐stored seed, while some ecotypes also resprout post‐fire. We aimed to improve understanding of the ecology and utilization of the species and its ecotypes in the wild. We surveyed 45 populations of wild rooibos across the species' range, distinguishing four ecotypes, and assessing their environmental preferences, density, demographics, extent, and effects of harvesting and fire on population health. Populations appeared demographically healthy with low incidences (average 5%) of mortality and stressed plants (9%). Reseeder and resprouters recruited equally from seed (seedlings comprised 4% of populations) and both exhibited wide‐ranging population densities (25 to 30,000 plants.ha^−1^). Population densities were higher where fires were more frequent and in younger post‐fire vegetation. Seedlings occurred in vegetation of all ages, implying some inter‐fire recruitment. The mean fire return period in surveyed populations was long (26 years) by fynbos standards (10–20 years), but rooibos persisted well in old vegetation suggesting that fires at high or low frequency do not pose significant threats to the species. Generally, harvesting levels were low at landscape and population scales; < 45% of sites on private land were subject to harvesting and there < 50% of plants showed evidence of harvesting. Illegal and overharvesting were uncommon (< 3% of sites). Population health and plant vigor were mostly unaffected by harvesting, suggesting that harvesting does not presently have large‐scale detrimental effects on wild rooibos.

## 1 Introduction

*Aspalathus linearis* (Burm.f.) Dahlg., or 'rooibos' (family Fabaceae), is a highly polymorphic perennial shrub endemic to the hyperdiverse Cape Floristic Region of South Africa (Takhtajan 1986). The species has a narrow geographic range, largely confined to the drier montane areas of the winter‐rainfall, northwestern part of the Fynbos Biome (Dahlgren 1968, 1988; Lötter and Le Maitre 2014). Rooibos is commercially cultivated on a large scale to produce rooibos tea, a traditional herbal health drink (Morton 1982) which is marketed internationally. Rooibos tea is a well‐established industry with only a very small portion of the produce (0.001% of approx. 20,000 metric tons annually) coming from wild harvested populations (van Heerden et al. 2003), although prior to the 20th century, it was exclusively collected in the wild (Malgas et al. 2010). With the ongoing utilization of wild populations of the species, knowledge of its distribution, ecology, and the effect of harvesting is vital for the sustainable management of the resource.

**FIGURE 1:**
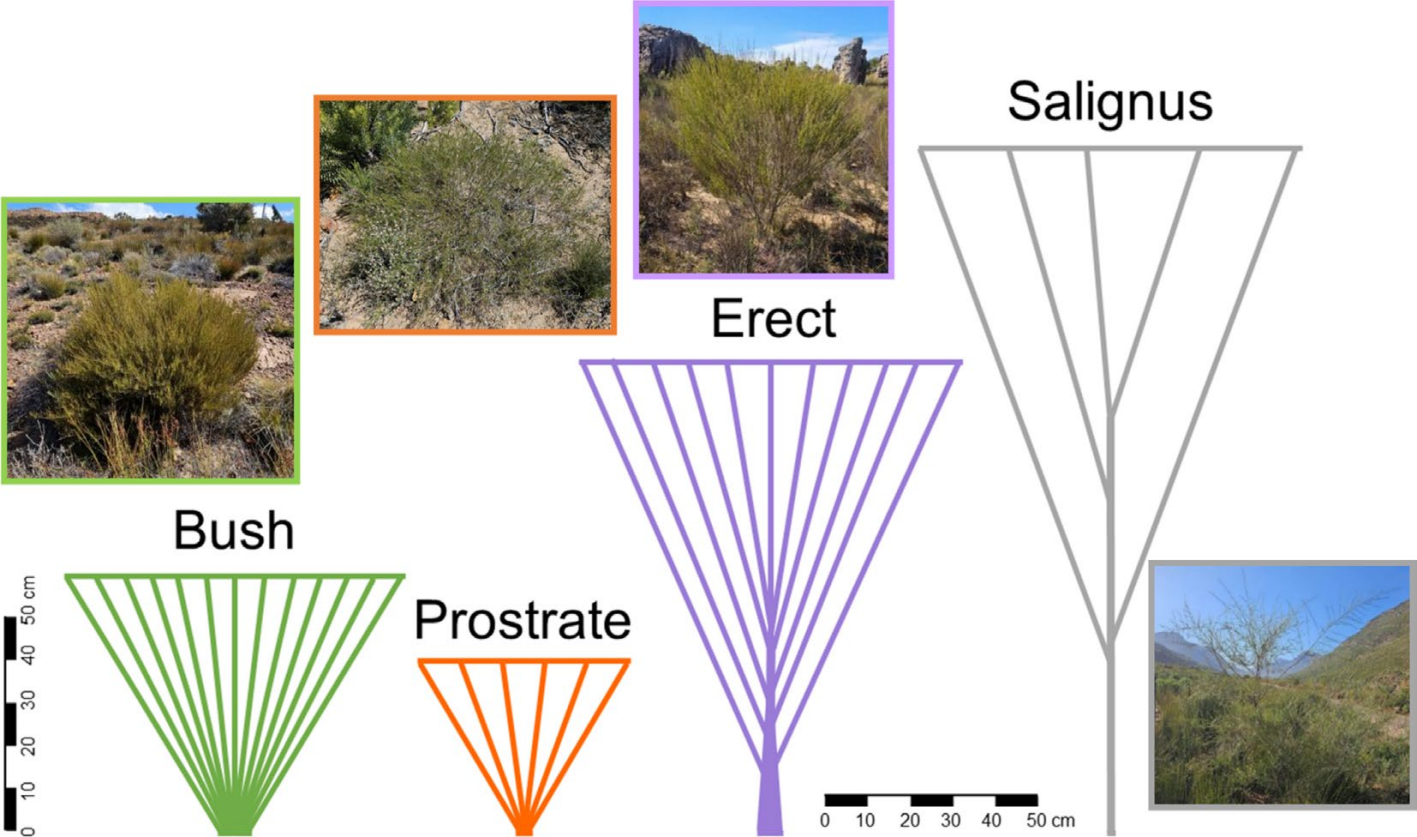
Photos of the ecotypes (Bush, Prostrate, Erect, Salignus) of *Aspalathus linearis* and schematics drawn to scale based on the median dimensions (of plant height, crown diameter, and basal diameter) recorded during population surveys.

Published information exists on aspects of rooibos' distribution, taxonomy, morphology (Dahlgren, 1986, Dahlgren 1988; Malgas et al. 2010; Lötter and Le Maitre 2014), cultivation (Hawkins et al. 2011; Bahramisharif et al. 2014; Smith and Hardie 2023), phenolic and phytopharmaceutical properties (van Heerden et al. 2003; Stander et al. 2017; Wilkinson et al. 2024), and genetic variation (van der Bank et al. 1995, 1999; Brooks et al. 2021). Rooibos occurs in fynbos, which is a tree‐less, sclerophyllous shrubland characterized by harsh conditions of summer heat and drought, gale force winds, cold wet winters, and recurrent crown‐consuming fires (Allsopp et al. 2014; Kraaij and van Wilgen 2014). The species grows along a north–south gradient of increasing annual rainfall of < 200 to > 500 mm (Malgas et al. 2010; Hawkins et al. 2011) on nutrient‐poor, sandy, acidic soils (Chimphango et al. 2016; Smith et al. 2018) derived from sandstone, quartzite, or shales. The plant takes the form of an erect to spreading, highly variable shrub or shrublet up to 2 m high with small needle‐like leaves. Flowers appear in spring to early summer, and the fruit is small lance‐shaped pods that usually contain one or two hard seeds (Dahlgren 1968) that are dispersed by ants and stimulated by fire to germinate in early winter (Cocks and Stock 1997). Some forms of rooibos can regenerate after fire by reseeding and resprouting. Reprouting forms can regenerate from subterranean lignotubers, whereas nonsprouting forms are killed by fire and re‐establish from seeds, with fire stimulating prolific germination (van der Bank et al. 1999). Rooibos is a N_2_‐fixer in the post‐fire environment, persisting in abundance for < 5 years (Cocks and Stock 2001), although plants as old as 20 years exist (Morton 1982). The species is exceptionally polymorphic and distinct ecotypes are recognized that differ genetically, in fire‐survival strategy, vegetative and reproductive morphology, enzymes, and flavonoids (van der Bank et al. 1995, 1999; van Heerden et al. 2003; Malgas et al. 2010). In this assessment, we distinguished four types (Figure 1), namely the Bush and Prostrate types, which are resprouters and wider than they are tall; and the Erect and Salignus types, which are obligatory reseeders with more upright stature (van Heerden et al. 2003; Malgas et al. 2010).

Harvesting of wild rooibos generally takes place once‐off in summer or autumn (December–May, but mostly March–April) every second year if sufficient growth renders the effort profitable. Leaves and twigs are cut by hand sickle at least 2 cm above previous cuts, removing < 70% of the volume of a plant. Harvesting communities and co‐operatives generally adhere to sustainable harvesting protocols (outlined by Malgas and Oettlé 2007) and organic certification schemes which ensure regulation of the industry. Overall, the population ecology of rooibos as a species, and moreover, its respective ecotypes, is inadequately understood, while the extent and effects of its utilization have not been coherently assessed.

To address some of these gaps, the current study presents components of an unpublished resource status assessment for wild rooibos (DFFE/SANBI 2024a) that was commissioned by the South African government. Specifically, we aim to (i) characterize wild rooibos populations in terms of their environmental preferences, density, demographics, health, and extent of harvesting; and (ii) assess the effects of harvesting, fire, and other physical factors on population health and plant size. Importantly, this assessment treats rooibos as a species in its entirety as well as recognizing four distinct ecotypes (Figure 1), respectively. A related study (Kraaij et al. n.d.) considered the species and its ecotypes' current occurrence, potential distribution, population size, and threats to its persistence. Jointly, the baseline data presented in these studies are intended to inform conservation status assessments (e.g., using IUCN Red List criteria), sustainability assessments (e.g., using the Non‐Detriment Finding process used by Parties to CITES) (Leaman and Oldfield 2014), and long‐term monitoring programs aimed at tracking the quantity and quality of this biotrade species over time.

## 2 Methods and Materials

### 2.1 Data Collection

Population surveys (*n* = 45) were undertaken during September 2023 to February 2024 across a large part of the geographic range of rooibos (Supporting Information S1) and across various land holder types, harvesting regimes, and post‐fire vegetation ages (3 to > 25 years). Given that prior information was not available on the species' occurrence across categories of land holding, harvesting, and post‐fire ages, survey sites were selected to be representative of the relative incidence of the species across these categories and representative of the relative abundance of the respective ecotypes.

At each survey site, belt transects of 2 m wide and of variable length were positioned a minimum of 5 m apart across a population of wild rooibos, until approximately 50 individual plants were recorded, taken as representative of that population. The total length of the transects surveyed was recorded to enable calculation of the density of plants in the population surveyed. In the case of very small or sparsely distributed populations, where the belt transect approach would be inefficient, the entire population (or up to 50 individuals) was surveyed systematically by temporarily marking plants that have already been recorded. In such cases, the size of the area surveyed was measured afterward in GIS to enable density calculations. The mean (± SE) length of transects surveyed per population was 87 (± 4) m and the mean (± SE) surface area surveyed per population was 1194 (± 35) m^2^. In each transect, comprehensive data on transect, habitat, and plant attributes were systematically collected as detailed in Table 1.

**TABLE 1:** Transect, habitat and plant attributes for which data were collected during wild rooibos population surveys.

Data type	Attribute	Description
Transect and habitat attributes	Geolocation	Coordinates of starting location of transect
	Surveyor	Name of surveyor(s)
	Date	Date of survey
	Property name	Farm/reserve name
	Transect identifier	Unique identifier
	Land holding	Private, Communal, Protected area on state land
	Soil type	Sand, Loam, Sand & gravel, Sand & rock, Loam, Clay
	Slope	Flat, Gentle, Moderate, Steep
	Aspect	Warm including W‐, NW‐, N‐ & NE‐facing slopes; or Cool including SW‐, S‐, SE‐ & E‐facing slopes
	Ecotype/growth form	Bush/Shrub, Prostrate, Erect/Upright/Tree, Salignus
	Life history strategy	Resprouter, Reseeder
	Vegetation canopy cover	Estimated as %
	Vegetation age	Years post fire (estimated in field, and verified later from fire history maps)
	Threats	Overharvesting, Invasive alien plants, Overgrazing, Land clearing, No obvious threats, Other (then note)
	Invasive alien plants	Note the species and estimate cover
	Harvest regime	Harvested regularly, Harvested occasionally, or Not harvested, based on information obtained from land owners, managers or harvesters
	Year last harvested	Estimate
	Percentage of population flowering	Estimated as % after having completed the recording of plant attributes
	Flower keel color	Yellow, Deep purple/red, Light purple/red, Brownish
	Transect or whole population surveyed	Select either
	Transect length	Recorded in meters
	Other plants present	List genera/species
	Other notes	
Plant attributes (recorded for each of 50 individuals)	Plant number	Sequentially numbered
	Status	Alive, Dead, Stressed (substantial leaf loss; partial die‐back such as of branch tips; and/or signs of pathogenic infestations), Seedling (defined as a small plant ≤ 30 cm high, with visibly young growth, and mostly single‐stemmed)
	Crown density	Dense, Intermediate, Sparse
	Plant height	Measured to the nearest 10 cm
	Crown diameter (at its widest)	Measured to the nearest 5 cm
	Basal diameter (at its widest)	Measured to the nearest cm
	Harvesting status	Harvested, or Not harvested, as determined from evidence of harvesting (cut stems) that remain visible on plants for up to 5 years post harvesting
	Growth since last harvest	Measured in cm
	Geolocation	Coordinates (automatically recorded)

### 2.2 Data Analysis

Basic descriptive statistics were used to characterize the biophysical, ecological, and management features of population survey sites. For each population, we calculated the incidence, as a percentage of the total number of plants surveyed in the population, of: dead plants, stressed adult plants (defined in Table 1), seedlings, and plants showing evidence of harvesting. Assessment of population (or site) characteristics relied on non‐parametric statistical analysis procedures, given that the relevant variables of population health and site characteristics were not normally distributed, and replication was low (*n* = 45). Kruskal–Wallis *H*‐test or Mann–Whitney *U*‐test was used to assess if the population density and the incidences of harvested plants, dead plants, stressed plants, and seedlings differed among: (i) ecotypes, (ii) slopes with cool vs. warm aspects, and (iii) resprouters vs. reseeders. With Spearman rank order correlation, we assessed the relationships between harvesting incidence, population health metrics, population density, fire frequency (the number of fires in 24 years), and vegetation post‐fire age.

To assess if plant size measures (plant height, crown diameter, and basal diameter; *n* = 2082) differed among the ecotypes, we used the Kruskal–Wallis *H*‐test followed by Dunn's multiple comparisons as these variables were not normally distributed. To assess relationships among plant size measures and post‐fire vegetation age for the species and ecotypes, respectively, we used Spearman rank correlation. Population demographics were assessed by histograms of size (in terms of crown diameter and height) frequency distributions at different post‐fire age classes (0–7 years, 8–20 years, and > 20 years post fire).

To assess the effects of biophysical factors and harvesting on the size of *A. linearis* plants, generalized linear models (Gamma family with logit link) were employed for the species and ecotypes. The respective measures of plant size, namely crown diameter, plant height, and basal diameter, were assessed in relation to the predictor variables, namely ecotype (for the species‐wide model), incidence of harvesting (% plants in population showing evidence of past harvesting), post‐fire veld age, and slope aspect (cool or warm). The steepness of slopes was not included as most sites occurred on gentle slopes (see Results). Fire frequency was omitted from analyses as it was negatively collinear with post‐fire veld age (Spearman rank correlation coefficient of −0.79, where < 0.60 is regarded as a threshold for inclusion of both factors into a model; Tabachnick and Fidell 1996). Crown diameter and basal diameter were square‐root transformed on account of right‐skewed distributions, while plant height was not transformed as model diagnostics exhibited good fit. Statistica v14.1.0. (TIBCO 2023) and the *Rcmdr* package in R Statistical Software v4.3.1 (R Core Team 2023) were used for statistical analyses.

## 3 Results

### 3.1 Population Attributes

**FIGURE 2:**
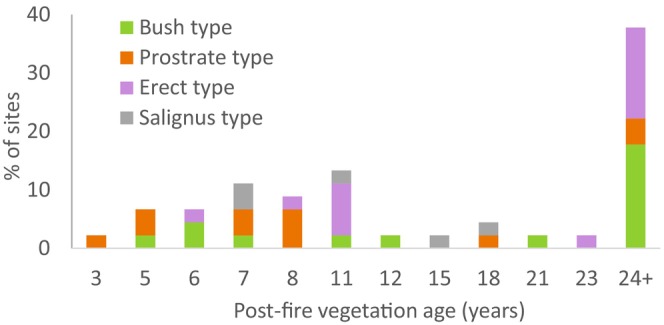
The incidence of survey sites and ecotypes across post‐fire vegetation ages.

We recorded 2082 plants in 45 populations, with most survey sites (69%) on private property and fewer sites on communal land (18%) and state protected land (13%) (Supporting Information S1). The populations occurred across 10 different national vegetation types (Rebelo et al. 2006) and most commonly in Cederberg and Bokkeveld Sandstone Fynbos (Supporting Information S1 and S2). Total vegetation canopy cover ranged from an estimated 40%–90% (median 70%), with *A. linearis* commonly associated with species of Restionaceae, Proteaceae, Mesembryanthemaceae, *Dodonaea* (Sapindaceae), *Metalasia, Eriocephalus*, and *Helichrysum* (Asteraceae), *Nylandtia* (Polygalaceae) and *Searsia* (Anacardiaceae). Most of the survey sites (84%) occurred on gentle to moderate slopes. The substrate at all survey sites comprised sand or loamy sand with various quantities of gravel or rock. Fifty‐eight percent of populations comprised resprouters (33% Bush and 25% Prostrate types) and 42% reseeders (31% Erect, 11% Salignus) (Supporting Information S1). The post‐fire vegetation age of sites at the time of surveying ranged from 3 years to > 24 years (median 12 years; Figure 2). Thirty‐eight percent of sites did not experience a fire during the 24 years preceding the surveys, while 40% of sites experienced one fire, 15% experienced two fires, and 7% experienced three fires, translating into an average fire return interval of ca. 26 years across the survey sites over the past 24 years.

### 3.2 Extent of Harvesting

According to land managers, 45% of the surveyed sites were subject to regimes of occasional or regular harvesting, respectively, while 55% had no harvesting. However, when considering whether individual plants showed evidence of prior harvesting (cut stems remain visible for up to 5 years) at the time of the survey, harvesting was not evident in any of the surveyed plants at some sites with regimes of harvesting. Overall, at sites with harvesting regimes, only 46% of plants showed evidence of harvesting. This finding implied that high, or even moderate, levels of harvesting should not be presumed for properties where harvesting regimes prevail as, on average, less than half of the plants get harvested there. Further analyses of the effects of harvesting on plant vigor thus considered evidence of harvesting on individual plants rather than the harvesting regime according to managers. At sites with a regime of no harvesting, 5% of plants showed evidence of harvesting. This included illegal harvesting at one of the six sites, which was on state protected land where harvesting is not permitted. Here the harvesting intensity was high with 63% of the population harvested.

About a third (31%) of the individuals of the Bush and Erect types showed evidence of harvesting, 20% of the Salignus type, and only 4% of the Prostrate type. The percentage of harvested individuals per population did not differ significantly among the ecotypes (Table 2). Neither did the incidence of harvesting differ between populations of resprouters vs. reseeders, nor between sites with cool vs. warm aspects (Table 2). The incidence of harvesting in populations was not correlated with fire frequency or with the vegetation age post fire (Table 3).

**TABLE 2:** Kruskal–Wallis *H*‐ or Mann–Whitney *U*‐test results for comparisons of the incidence of harvested plants, stressed adults, dead plants, seedlings, and the population density among the ecotypes (Bush, Prostrate, Erect, Salignus) of *Aspalathus linearis* , resprouters vs. reseeders, and slopes with cool vs. warm aspects (*n* = 45 populations). Statistically significant differences (*p* < 0.05) are in red.

Population metric	Ecotype	Aspect cool vs. warm	Resprouter vs. Reseeder
% Harvested	*H*_3,41_ = 1.87, *p* = 0.600	*U*_43_ = 168.0, *p* = 0.084	*U*_43_ = 215.5, *p* = 0.437
% Stressed adults	*H*_3,41_ = 1.64, *p* = 0.650	*U*_43_ = 232.5, *p* = 0.817	*U*_43_ = 223.5, *p* = 0.549
% Dead plants	*H*_3,41_ = 9.69, *p* = 0.021	*U*_43_ = 213.0, *p* = 0.494	*U*_43_ = 140.0, *p* = 0.011
% Seedlings	*H*_3,41_ = 3.23, *p* = 0.357	*U*_43_ = 227.0, *p* = 0.720	*U*_43_ = 219.0, *p* = 0.490
Population density	*H*_3,41_ = 2.28, *p* = 0.517	*U*_43_ = 213.0, *P* = 0.494	*U*_43_ = 249.0, *p* = 0.991

**TABLE 3:** Spearman rank order correlation coefficients for the relationships between the incidence of harvesting, different metrics of population health, the population density of *Aspalathus linearis* , fire frequency (the number of fires in 24 years), and the post‐fire age of the vegetation. Statistically significant correlations (pairwise 2‐sided *p* < 0.05) are shown in red.

	% Harvested	% Dead plants	% Stressed adults	% Seedlings	Population density	Fire frequency	Vegetation age post fire
% Harvested		0.28	0.08	−0.26	−0.08	−0.06	0.06
% Dead plants	0.28		0.32	−0.29	0.12	0.10	−0.07
% Stressed adults	0.08	0.32		−0.13	0.05	0.04	0.12
% Seedlings	−0.26	−0.29	−0.13		0.34	0.32	−0.32
Population density	−0.08	0.12	0.05	0.34		0.36	−0.48
Fire frequency	−0.06	0.10	0.04	0.32	0.36		−0.79
Vegetation age post fire	0.06	−0.07	0.12	−0.32	−0.48	−0.79	

### 3.3 Population Health

The density of *A. linearis* plants ranged widely from 25 to almost 30,000 plants per hectare (median 839 plants/ha; mean 3019). On average, 83.4% ± 2.2% (mean ± SE; range 2%–83%) of individuals in populations were healthy, while 8.5% ± 4.0% (range 0%–38%) appeared stressed and 4.6% ± 5.9% (range 0%–79%) were dead. Seedlings comprised 3.5% ± 5.2% (range 0%–59%) of populations. These metrics of population health, population density, and incidence of seedlings did not differ between ecotypes or between cool and warm slopes (Table 2). Neither did these metrics differ between populations comprised of resprouters vs. reseeders, other than that mortality was significantly higher in reseeder populations (Table 2). There was thus no evidence of reseeders being more successful than resprouters at recruiting from seed, or that population densities differed between resprouters and reseeders. There was a statistically significant difference among the ecotypes in the incidence of dead plants, but post hoc pairwise comparisons did not show significant differences among particular pairs of ecotypes. The incidences of stressed and dead plants were positively correlated (Table 3). The incidence of seedlings was positively correlated with the population density of rooibos, and both these measures were positively correlated with fire frequency and negatively correlated with vegetation age post fire (Table 3). Populations were thus denser and had relatively more seedlings in vegetation that burnt more frequently and in vegetation of younger post‐fire ages.

### 3.4 Plant Allometry and Population Demographics

On average, *A. linearis* plants (*n* = 2082) were 87 cm high (coefficient of variation, CV, of 71%), with a crown diameter of 89 cm (CV 64%) and a basal diameter of 8 cm (CV 118%). The dimensions of the ecotypes differed significantly in terms of plant height (*H*_3,2078_ = 1287.6, *p* < 0.001), crown diameter (*H*_3,2078_ = 188.1, *p* < 0.001), and basal diameter (*H*_3,2078_ = 309.4, *p* < 0.001) (Supporting Information S3). The Salignus type, and thereafter the Erect type, were the tallest, the Bush type had the largest basal diameter, while the Prostrate type was overall the smallest (shortest with the smallest crowns). The Bush and Erect types had the densest crowns, whereas the Prostrate type had intermediate crown densities and the Salignus type virtually exclusively sparse crowns (Supporting Information S4). Based on these findings, archetypal growth forms were differentiated and schematically illustrated (Figure 1). Considering the allometric relations of all rooibos plants (ecotypes jointly), crown diameter was best correlated with other measures of plant size (basal diameter and plant height) and with vegetation post‐fire age (Supporting Information S5). Plant height and basal diameter were poorly correlated, likely due to the different forms of the ecotypes and large variation (CV 118%) in basal diameter. Within ecotypes, the allometric relations were tighter, particularly in the Bush type, which showed good correlation of crown diameter with basal diameter and plant height. Relationships within the respective ecotypes between post‐fire vegetation age and measures of plant size were not tight.

**FIGURE 3:**
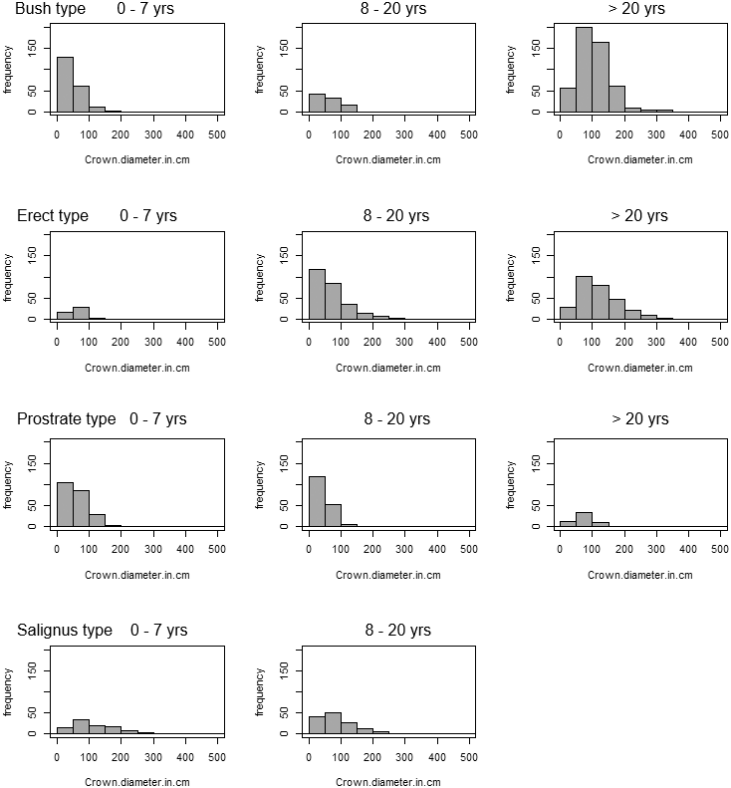
Crown size frequency distributions of the ecotypes (Bush, Prostrate, Erect, Salignus) of *Aspalathus linearis* with increasing vegetation age post fire (categorized as 0–7 years, 8–20 years, and > 20 years of post‐fire age; in respective columns).

**FIGURE 4:**
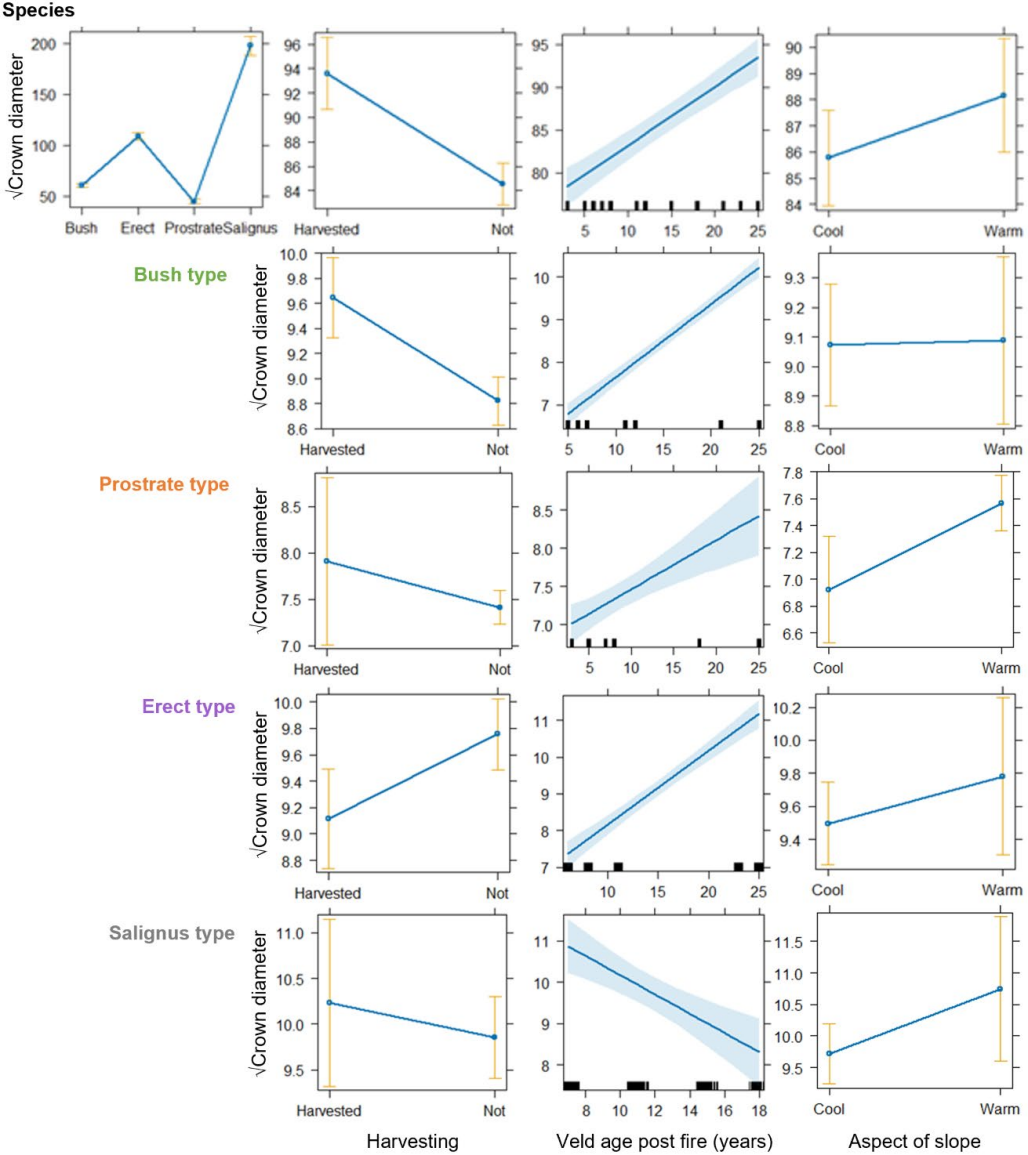
Effects of ecotype, harvesting, vegetation age post fire, and the aspect of slopes on the square root of crown diameter of *Aspalathus linearis* plants. Results are shown for the species and for the ecotypes respectively, based on the model outputs in Table 4. Shaded bands depict standard errors and whiskers 95% confidence intervals.

Assessment of size class frequency distributions (in terms of crown diameter (Figure 3) and plant height (Supporting Information S6)) at different post‐fire age classes displayed healthy population demographics with relatively abundant small (~young) plants at early post‐fire ages and increases in the number of larger individuals with increasing time post fire. Distributions mostly remained bell‐shaped in the oldest vegetation age class (≥ 20 years), indicating the continued presence of small plants in old vegetation. Due to the unequal survey effort across the different vegetation ages (see data distribution along x‐axes of vegetation age panels in Figure 4), interpretation of these histograms should take account of their shapes (relative frequencies) rather than the absolute frequencies of plants in the size‐ and vegetation age class categories. For instance, few plants of the Erect type were surveyed in the 0–7‐year age class compared to the numbers of plants in older classes, which does not suggest that numerous plants recruited at ages of ≥ 8 years. These size‐frequency distributions corroborate that plant size does not increase linearly with post‐fire age, as the Bush and Erect types continued to increase in crown size and height with time post fire, while it was less evident in the Prostrate and Salignus types (Figure 3; Supporting Information S6). The size‐frequency distributions furthermore indicate that plants generally persist, and some ecotypes continue to grow in size, in old vegetation.

### 3.5 Effects of Fire and Harvesting

Plant size mostly increased with veld age post fire (Table 4; Figure 4; Supporting Information S7 and S8), except in the Salignus ecotype, which decreased in all dimensions, and the Prostrate type, which decreased in height with age post fire. The Spearman rank correlations further showed that the incidence of harvesting was not correlated with any of the measures of population health that we investigated, that is, the incidence of seedlings, dead, or stressed plants. Neither was the incidence of harvesting correlated with population density. The generalized linear models assessing effects of harvesting on plant size (~vigor) showed (Table 4) that crown diameter was larger in harvested plants of the species as a whole and in the Bush type, unaffected by harvesting in the Prostrate and Salignus types, but that crowns were smaller in harvested plants of the Erect type (Figure 4). Harvested plants of *A. linearis* were taller than those that were not harvested, except in the Salignus type, where harvested plants were shorter (Supporting Information S7). Basal diameter was larger in harvested plants of the species as a whole and in the Erect and Salignus types, but unaffected in the Bush and Prostrate types (Supporting Information S8).

**TABLE 4:** Estimates of linear models that assessed different dimensions of plant size (the square‐root of plant height, crown diameter, and basal diameter, respectively) of *Aspalathus linearis* in relation to ecotype (Bush, Prostrate, Erect, Salignus), incidence of harvesting, veld age post fire, and the aspect of slopes. Models were run for the species in totality, and for the ecotypes respectively. Effects plots are shown in Figure 4 and Supporting Information S6 and S7.

	Ecotype prostrate^a^	Ecotype erect^a^	Ecotype salignus^a^	Harvesting^b^	Vegetation age post fire	Aspect warm^c^
Species (*n* = 2082)						
Plant height	−15.68****	48.35****	137.45****	9.05****	0.69****	2.39**
√Crown diameter	−0.21	0.73****	2.26****	0.35**	0.15****	0.39***
√Basal diameter	−0.11	−0.48****	−1.14****	0.32****	0.03****	−0.18***
Bush type (*n* = 787)						
Plant height				13.43****	1.08****	4.97***
√Crown diameter				0.82****	0.17****	0.01
√Basal diameter				0.03	0.04****	−0.79****
Prostrate type (*n* = 454)						
Plant height				1.65	−0.32****	−10.04****
√Crown diameter				0.50	0.06****	0.65***
√Basal diameter				−0.14	< 0.01	−0.45**
Erect type (*n* = 601)						
Plant height				2.67	0.43*	3.69
√Crown diameter				−0.64***	0.20****	0.29
√Basal diameter				0.22**	0.05****	0.18*
Salignus type (*n* = 240)						
Plant height				−53.67****	−4.37****	44.74**
√Crown diameter				0.38	−0.23****	1.03
√Basal diameter				0.20**	−0.07****	−0.21**

* *p* < 0.1.

** *p* < 0.05.

*** *p* < 0.01.

**** *p* < 0.001.

^a^ Compared against the Bush type as reference category.

^b^ Compared against no harvesting as reference category.

^c^ Compared against cool aspect as reference category.

## 4 Discussion

### 4.1 Population Ecology

Large variation in size and form was observed among individuals of *A. linearis* and within ecotypes. Despite this variation, archetypal growth forms could be differentiated, which generally agreed with the characterization of ecotypes by van Heerden et al. (2003) and Malgas et al. (2010). Our assessment of allometric relations suggested that crown diameter and plant height are the most representative measures of plant size suitable for use in further monitoring endeavors. Poor correlations between measures of plant size and post‐fire age, as well as our evaluation of size‐frequency distributions, indicated that linear increases in plant size with post‐fire age should not be assumed in *A. linearis* or its ecotypes, and that post‐fire vegetation age should be considered a discrete factor in assessments of plant vigor.

Populations of wild rooibos collectively, and of the respective ecotypes, appeared demographically healthy with low incidences of mortality and stressed plants. The density of wild rooibos plants ranges widely, and at the higher end of this spectrum, compares well to that in cultivated fields, where 8000–16,000 seedlings are planted per hectare of which usually < 50% survive (Pretorius G, 2024, pers. obs.). Reseeder and resprouter ecotypes were equally successful at recruiting from seed and exhibited comparable population densities. Population densities and the incidence of seedlings were higher where fires were more frequent and in younger post‐fire vegetation, in accordance with what is known for nitrogen‐fixing *Aspalathus* species that are early post‐fire colonizers exhibiting heat‐stimulated germination (Cocks and Stock 1997, 2001). However, mortality was not higher in older vegetation, and plants exhibited considerable longevity, which contrasts with the notion that rooibos does not persist in abundance beyond 5 years after fire (Cocks 1994; Cocks and Stock 2001). Seedlings and young (~small) plants occurred in vegetation of all ages, suggesting a low level of inter‐fire recruitment. Alternatively, these small plants in old vegetation may be older plants that recruited shortly after fire but remained stunted. Given the space‐for‐time design of our assessment, results such as displayed in size‐frequency histograms represent single snapshots in time of different populations at various post‐fire ages. More rigorous assessment of population dynamics would require repeat surveys of aging populations over time.

The sizes of the surveyed populations, and the density of plants within, varied by several orders of magnitude, displaying a highly irregular, clumped, and scattered distribution across the landscape, with populations typically occupying only a small part of seemingly suitable, homogenous swathes of habitat. This has relevance for attempts at estimating the total population size and status of the species and its ecotypes (Kraaij et al. n.d.), and for other conservation actions focused on the resource.

### 4.2 Fire

The mean fire return interval (approx. 26 years) that the surveyed rooibos populations experienced during the 25 years preceding this study was remarkably long by fynbos standards (mean fire return intervals ranging between 10 and 20 years; Kraaij and van Wilgen 2014), generally, and compared to the median fire return interval of 13 years recorded for the Cedarberg Nature Reserve (van Wilgen et al. 2010) in the centre of rooibos' distribution range. Although population densities declined with post‐fire veld age, the decline was not drastic and populations commonly occurred in vegetation > 24 years old. Populations furthermore seem to persist in the same areas over long time periods, as confirmed by field verification of the species' presence at numerous historical locations (Kraaij et al. n.d.). Infrequent fires therefore do not appear to threaten the persistence of wild rooibos. On the other hand, changing fire regimes, and in particular, too frequent and too severe fires, threaten the persistence of biodiversity in the Fynbos Biome generally (Kraaij and van Wilgen 2014). However, too frequent fires do not seem common in the rooibos distribution range. The relatively arid fynbos in which rooibos occurs is unlikely to sustain high frequency fires that would negatively impact this fast‐maturing pioneer species. Furthermore, rooibos' myrmecochorous, soil‐stored seeds should be resilient to high severity fires (Bond and Slingsby 1983) although such fires might reduce resprouting vigor and survival of resprouting ecotypes (Moreno and Oechel 1993; Marais et al. 2014). Overall, inappropriate fire regimes do not currently appear to have significant negative impacts on *A. linearis* and are not expected to become a major threat to the species in the future.

### 4.3 Harvesting

Evidence from this study collectively suggests that harvesting is not currently having notable detrimental effects on wild rooibos. The incidence of harvesting was higher in the Bush and Erect types than in the Salignus and Prostrate types, which correlate with Malgas et al. (2010) noting that the former ecotypes are popular for harvesting purposes on account of high yields. The Bush and Erect types are thus most likely to suffer any potential negative effects of harvesting, while the Salignus and Prostrate types are least threatened. Besides the selection for particular ecotypes, harvesting appeared indiscriminate among resprouters and reseeders, sites with cool or warm aspects, in terms of vegetation post‐fire age, and population density. The latter suggests that denser populations do not necessarily elicit more complete harvesting of populations. The extent of harvesting recorded during our surveys (70% of which was on private land) was relatively low at various levels of observation, that is, more than half of the surveyed sites had regimes of no harvesting; less than half of the assessed plants subject to regimes of regular or occasional harvesting showed evidence of harvesting during the preceding ~5 years; and overharvesting or illegal harvesting was deemed a threat at only 13% of the survey sites, while evidence of illegal harvesting was observed at a single site.

Harvesting (at the intensity that prevailed in the surveyed populations) furthermore did not have consistent detrimental effects on measures of population health or plant size. On the contrary, harvested plants were sometimes larger in particular dimensions than unharvested plants. These counterintuitive results may be due to selection by harvesters for larger plants, as larger plants are easier to locate and offer higher yields. However, without a rigorous experimental design controlling for plant size prior to harvesting, the true reason for these findings cannot be established. The results do, however, suggest that harvesting at the intensity that prevailed in the surveyed populations mostly does not have major detrimental effects on the size of *A. linearis* plants, apart from reductions in the height of the Salignus type and in the crown size of the Erect type. Accordingly, harvesters, during informal discussions, commonly reported that they have harvested the same areas over the course of decades without it causing notable declines in the species' abundance or sustained reductions in yields. With wild‐harvested rooibos comprising only 0.001% of the total annual production of rooibos tea, the small demand for wild rooibos (induced by inherent marketing constraints and challenges that are unlikely to change in the near future) (Pretorius G, 2024, unpubl. data) furthermore greatly reduces the current pressure and arguably the potential threat that harvesting imposes on the species in the wild.

The occurrence of illegal and uncontrolled harvesting of wild rooibos in protected areas on state land, albeit limited, is concerning. Protected area authorities lack the resources to patrol the vast areas in which wild rooibos occurs naturally and are unlikely to be able to launch specific actions to protect wild rooibos specifically (Malherbe D, 2024, pers. comm.). Illegal harvesting on state land could potentially be addressed by introducing a permit system that would allow harvesting of wild rooibos subject to conditions that would ensure sustainability and facilitate monitoring of the resource. Although overharvesting or illicit harvesting on private land did not appear to be a significant issue, several existing or new initiatives can contribute to securing conservation of the resource into the future. These include (i) sustained adherence/subscription to established sustainable harvesting protocols (Malgas and Oettlé 2007) and certification schemes; (ii) adoption of a Conservation and Sustainable Use guideline for cultivated and wild rooibos, which currently is in draft form; (iii) development of a Rooibos Charter (in collaboration with South African government) intended to set a standard for evaluation of rooibos producers and harvesters; and (iv) employment of a Rooibos Biodiversity Plan for the Cederberg Indigenous People's Association that is aligned with the Khoikhoi Peoples' Rooibos Biocultural Community Protocol. Accordingly, levies paid by the rooibos industry in terms of Access and Benefit Sharing agreements could then be channeled to actions that would promote the conservation of the resource. Such actions may include the appointment of field rangers to legally enforce the policies outlined by the Biodiversity Plan, training of wild harvesters, and conducting monitoring of wild rooibos.

Our assessment of the effects of harvesting on wild rooibos was constrained by the availability of sites at the time of surveying, which represented random and variable harvesting regimes. Harvesting could not be quantified beyond noting, per plant, the presence or absence of any evidence of harvesting in the preceding ~5 years. More rigorous assessment of the effects of harvesting would require repeat surveys of sites pre‐and post‐harvesting or surveys of paired sites with and without harvesting. Ideally, the experimental design should control for pre‐harvesting plant size and harvesting intensity and frequency.

## 5 Conclusions

This study is the first to have assessed, throughout a large part of *A. linearis* range, basic aspects of the population ecology of the species and its respective ecotypes. We found that the species is not overly sensitive to too frequent or too infrequent fires; that the existing level of harvesting of wild rooibos is low; and that overharvesting and illegal harvesting are uncommon. Harvesting furthermore does not presently have obvious detrimental effects on population health or plant vigor. This national‐level assessment of wild rooibos should inform conservation status assessments (e.g., using IUCN Red List criteria), sustainable utilization by industry, and the development of relevant policy by regulators for this key biotrade species, in addition to having provided a dataset and monitoring approach to serve as a baseline for future assessment of the status or condition of the resource (DFFE/SANBI 2024b).

## Ethics Statement

The authors have nothing to report.

## Conflicts of Interest

The authors declare no conflicts of interest.

## Supporting information


**Data S1:** pei370079‐sup‐0001‐DataS1.docx.

## Data Availability

Data subject to third party restrictions—The data that support the findings of this study are available from the first author subject to permission from the South African National Biodiversity Institute (sanbi.org). Privacy and ethical restrictions apply to these data, which were sourced subject to confidentiality arrangements with landowners on whose properties Rooibos grows.
