# Supplementary material for: Population Ecology and Harvesting of Rooibos ( Aspalathus linearis ) and Its Ecotypes in the Wild, South Africa
Source: Plant Environ Interact. 2025 Aug 5;6(4):e70079. doi: 10.1002/pei3.70079 (PMC12325480; doi:10.1002/pei3.70079)
Supplement: Supplementary file 1 — Data S1: pei370079‐sup‐0001‐DataS1.docx. [file PEI3-6-e70079-s001.docx]

**Supplementary material**


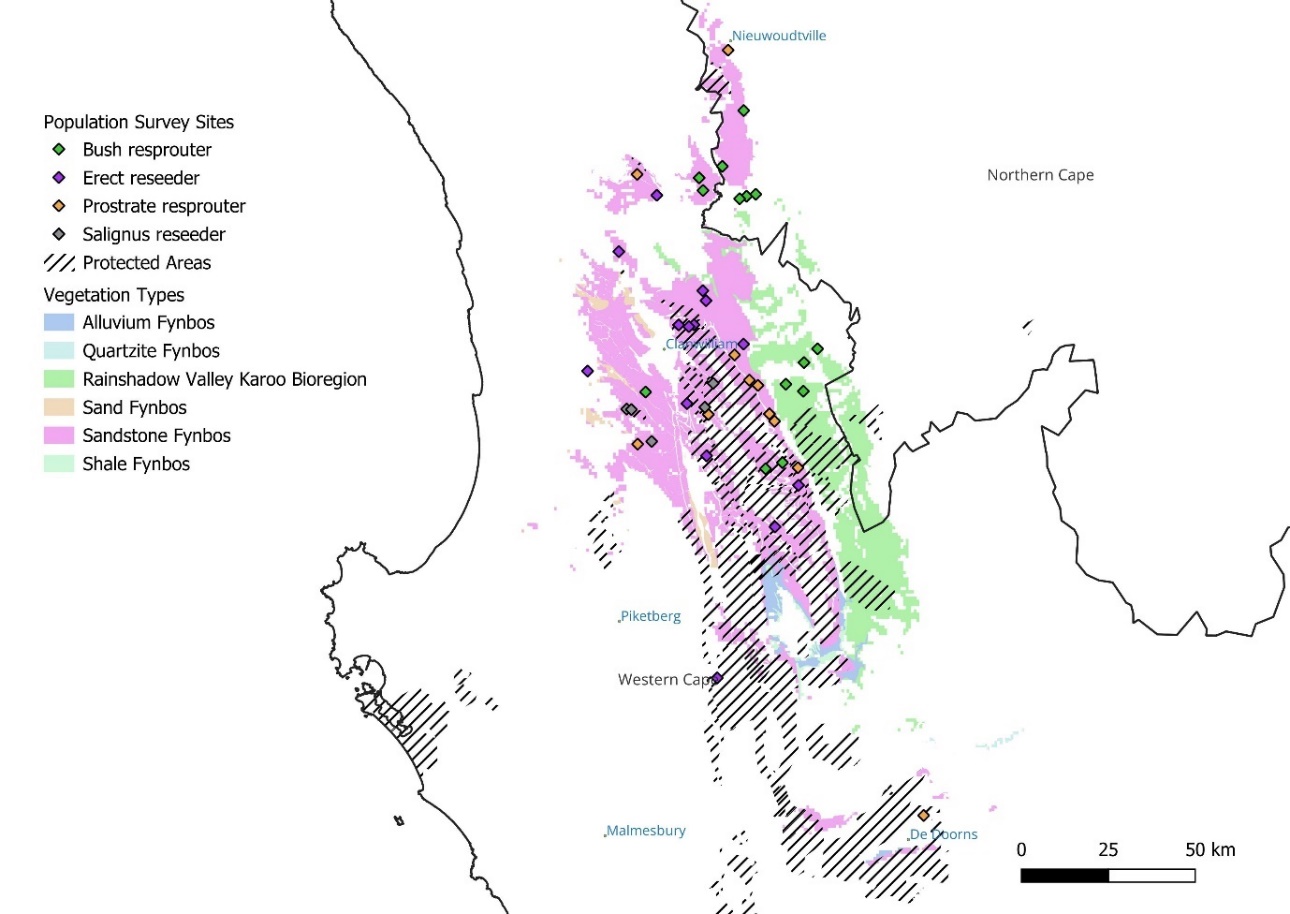


**Supplementary S1.** Locations of population survey sites (coloured in terms of the resident ecotype) in relation to *Aspalathus linearis*’ predicted distribution range (after Kraaij et al., submitted) shaded based on vegetation type, and the occurrence of proclaimed protected areas.

**Supplementary S2.** National vegetation types (after Rebelo et al., 2006) and their conservation status^Ɨ^ where population surveys were located.

| Vegetation Type | Number of survey sites/populations | Conservation status; Criterion* |
| --- | --- | --- |
| Cederberg Sandstone Fynbos | 19 | Not threatened |
| Bokkeveld Sandstone Fynbos | 6 | Not threatened |
| Olifants Sandstone Fynbos | 5 | Not threatened |
| Nardouw Sandstone Fynbos | 4 | Not threatened |
| Swartruggens Quartzite Fynbos | 3 | Not threatened |
| Graafwater Sandstone Fynbos | 2 | Not threatened |
| Agter-Sederberg Shrubland | 1 | Not threatened |
| Leipoldtville Sand Fynbos | 1 | Endangered; B1 |
| Northern Inland Shale Band Vegetation | 1 | Not threatened |
| Winterhoek Sandstone Fynbos | 1 | Not threatened |

*Criterion: B1 = high rates of habitat loss.

^Ɨ^ Source: DFFE. 2022. The revised national list of ecosystems that are threatened and in need of protection. National Environmental Management: Biodiversity Act (10/2004). Government Gazette No. 47526, Notice 2747, 18 November 2022. Department of Forestry, Fisheries and the Environment, South Africa.


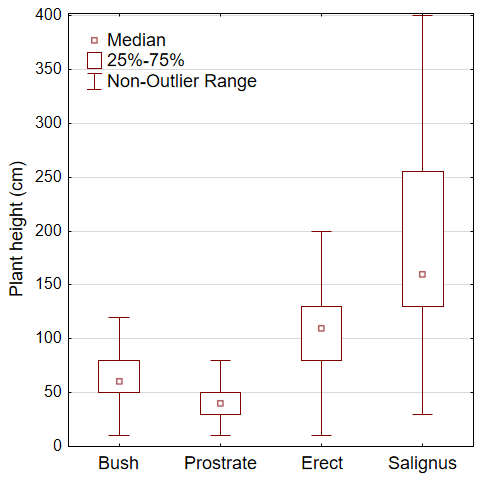

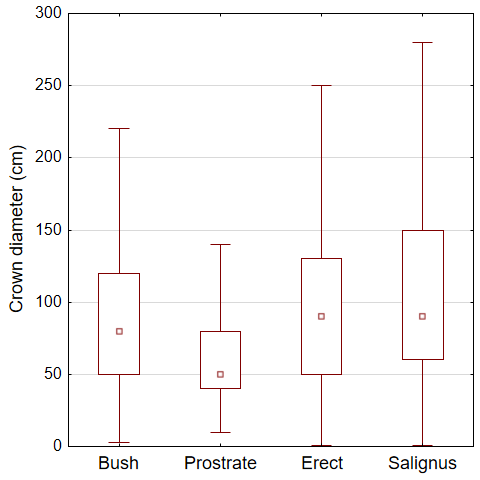

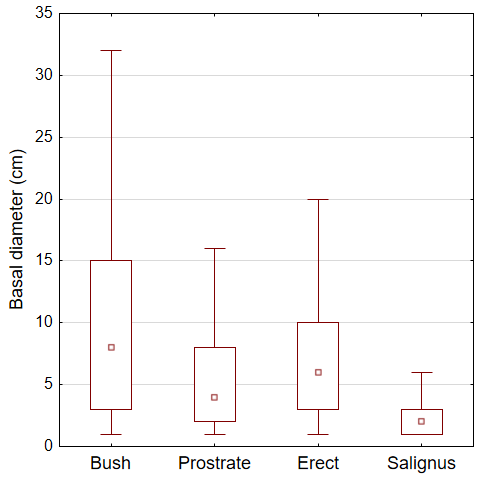


a

b

c

d

d

a

b

c

b

a

ac

c

A

B

C

**Supplementary S3.** Variation in plant size dimensions (A) plant height, (B) crown diameter, and (C) basal diameter across the ecotypes (Bush, Prostrate, Erect, Salignus) of *Aspalathus linearis*. Disparate small letters denote significant differences among ecotypes based on Kruskal Wallis *H* test results (see text) and Dunn’s multiple comparisons.

**Supplementary S4.** Variation in crown density across the ecotypes (Bush, Prostrate, Erect, Salignus) of Aspalathus linearis.

**Supplementary S5.** Spearman rank order correlation coefficients for the relationships between different dimensions of plant size and post-fire vegetation age for all plants of Aspalathus linearis combined, and for the different ecotypes respectively. Statistically significant correlations (P<0.05) are shown in red.

|  | Plant height | Crown diameter | Basal diameter |
| --- | --- | --- | --- |
| **All plants** |  |  |  |
| Crown diameter | 0.53 |  |  |
| Basal diameter | 0.05 | 0.56 |  |
| Vegetation age post fire | 0.26 | 0.44 | 0.29 |
| **Bush type** |  |  |  |
| Crown diameter | 0.68 |  |  |
| Basal diameter | 0.38 | 0.71 |  |
| Vegetation age post fire | 0.45 | 0.54 | 0.23 |
| **Prostrate type** |  |  |  |
| Crown diameter | 0.51 |  |  |
| Basal diameter | 0.03 | 0.26 |  |
| Vegetation age post fire | -0.06 | 0.09 | 0.23 |
| **Erect type** |  |  |  |
| Crown diameter | 0.44 |  |  |
| Basal diameter | 0.30 | 0.74 |  |
| Vegetation age post fire | 0.11 | 0.53 | 0.34 |
| **Salignus type** |  |  |  |
| Crown diameter | 0.61 |  |  |
| Basal diameter | 0.54 | 0.74 |  |
| Vegetation age post fire | -0.27 | -0.33 | -0.33 |

***
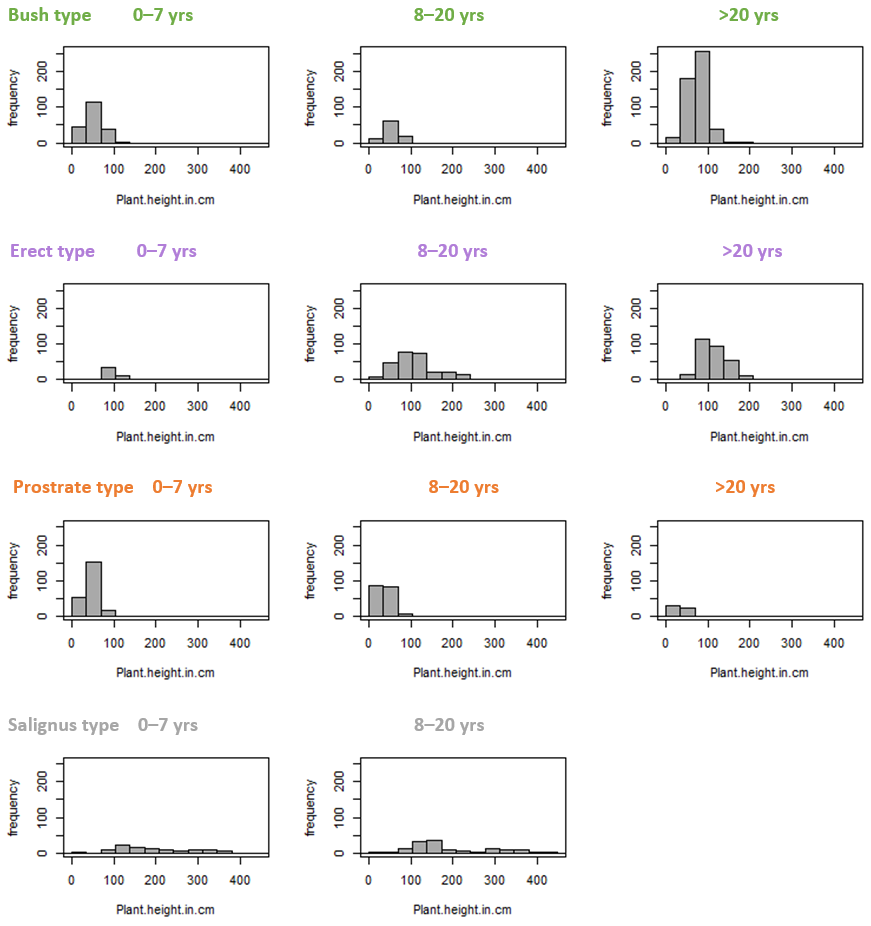
***

**Supplementary S6.** Plant height class frequency distributions of the ecotypes (Bush, Prostrate, Erect, Salignus; in respective rows) of Aspalathus linearis with increasing vegetation age post fire (categorised as 0–7 years, 8–20 years, and >20 years of post-fire age; in respective columns).


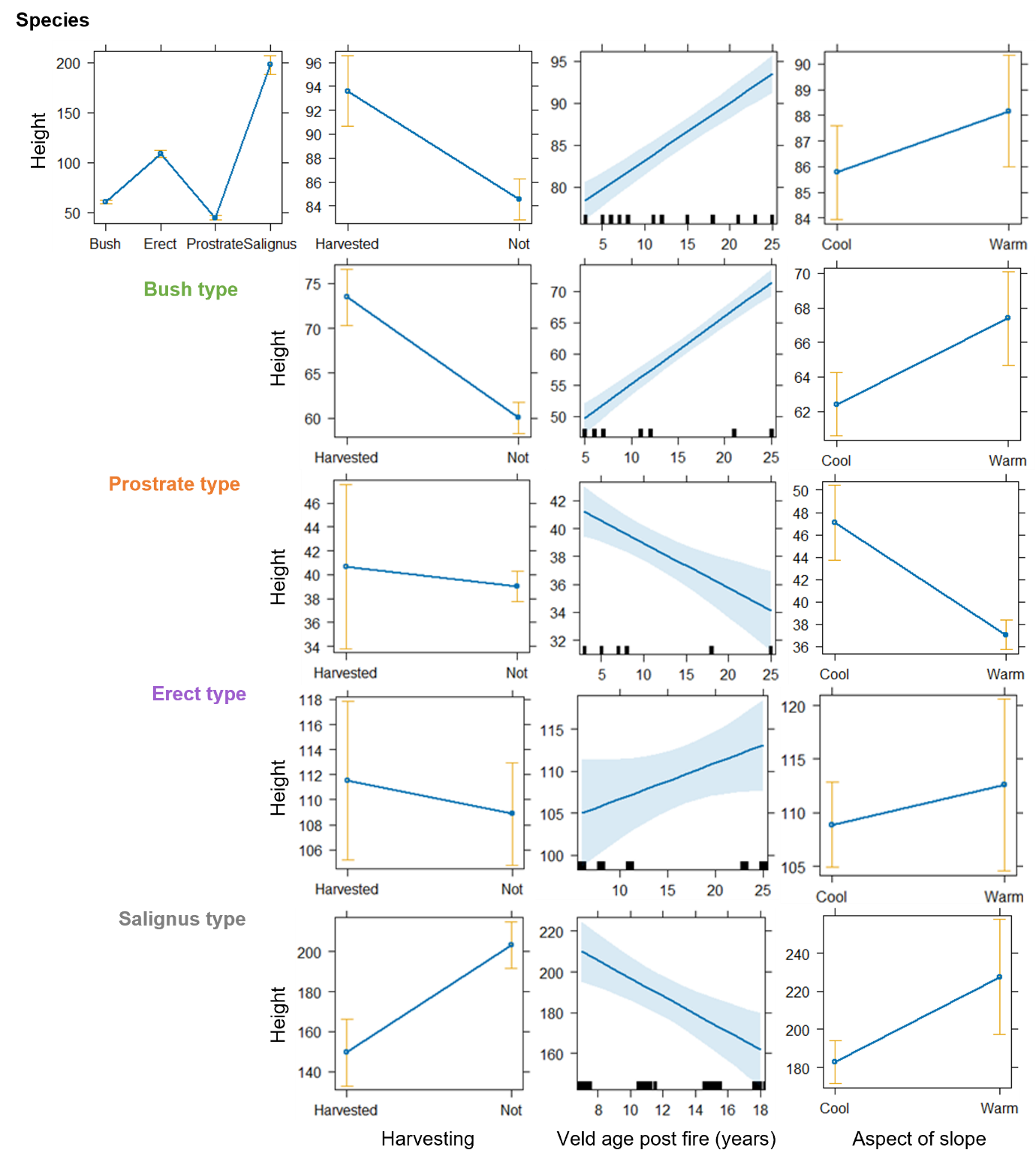


**Supplementary S7.** Effects of the factors ecotype, harvesting, vegetation age post fire, and the aspect of slopes on the height of Aspalathus linearis plants. Results are shown for the species and for the ecotypes respectively. Effects shown are based on the model outputs presented in Table 3. Shaded bands depict standard errors and whiskers 95% confidence intervals.


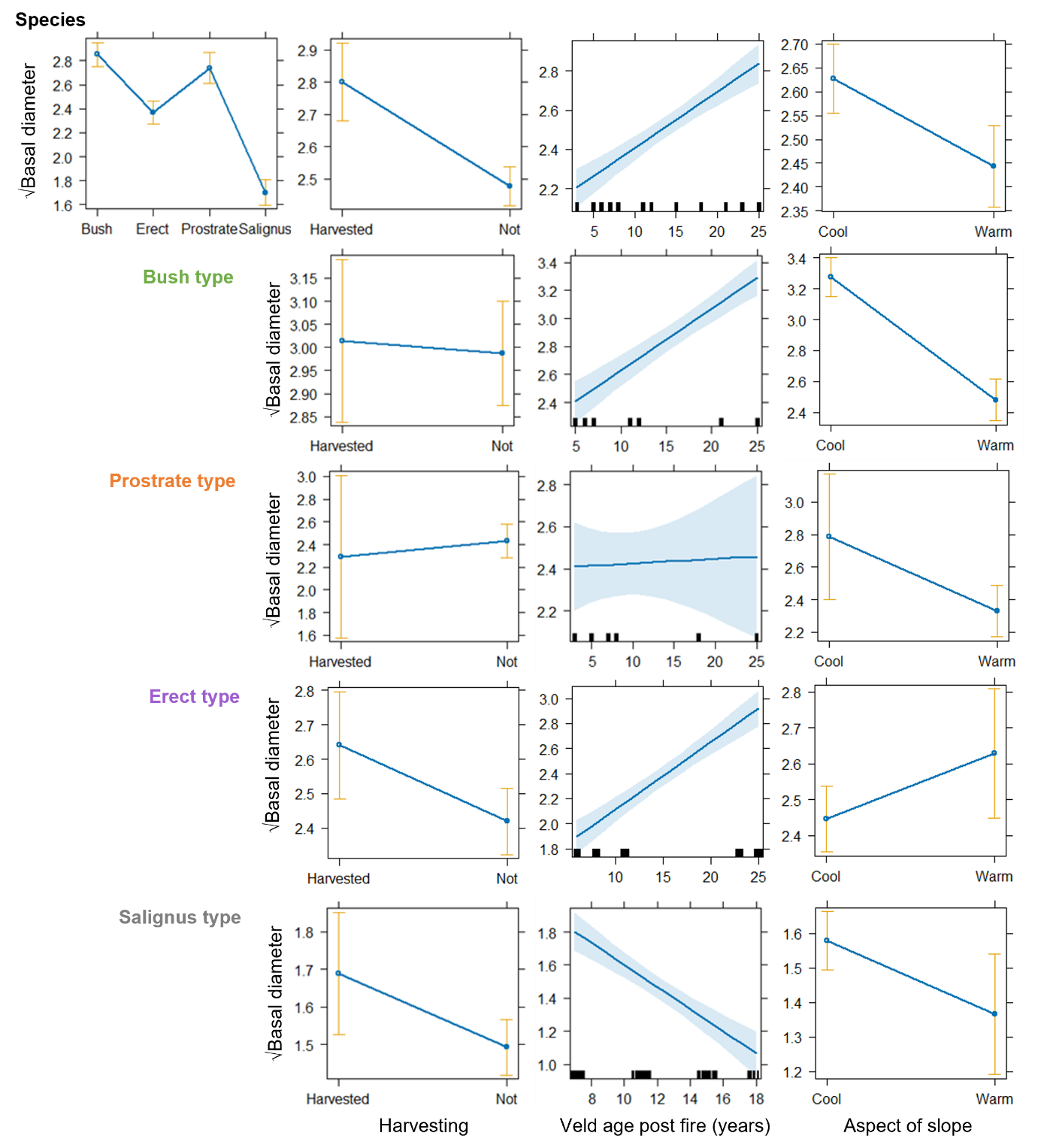


**Supplementary S8.** Effects of the factors ecotype, harvesting, vegetation age post fire, and the aspect of slopes on the square root of basal diameter of Aspalathus linearis plants. Results are shown for the species and for the ecotypes respectively. Effects shown are based on the model outputs presented in Table 3. Shaded bands depict standard errors and whiskers 95% confidence intervals.
